# The Use of Work-Home Practices and Work-Home Conflict: Examining the Role of Volition and Perceived Pressure in a Multi-Method Study

**DOI:** 10.3389/fpsyg.2019.02362

**Published:** 2019-10-18

**Authors:** Joni Delanoeije, Marijke Verbruggen

**Affiliations:** Work and Organization Studies, Faculty of Economics and Business, KU Leuven, Leuven, Belgium

**Keywords:** work-home practices, work-home conflict, telework, part-time work, preferences, volition, perceived pressure, vignette

## Abstract

In response to the rising number of individuals who have to combine work and home responsibilities, organizations increasingly offer work-home practices. These are HR-practices such as telework and part-time work that can help employees to combine work and home roles. However, extant research on the relationship between work-home practice use and both work-to-home conflict (i.e., work interfering with private life) and home-to-work conflict (i.e., private life interfering with work) shows inconsistent results. In this study, we posit that employees’ work-home conflict does not so much depend on whether or not they use a specific work-home practice, but rather on (1) the degree to which their (non-)use of this practice is in line with their preference (i.e., volition) and (2) the pressure they experience from the work and/or the home environment to act in another way than they prefer (i.e., perceived work pressure and perceived home pressure). Hypotheses are tested for two specific work-home practices (i.e., home-based telework and part-time work) in both a field study and an experimental between-subject vignette study. Results show that work-home conflict is affected by volition, perceived work pressure and perceived home pressure; yet, some differences were found between the two types of work-home conflict (i.e., work-to-home and home-to-work conflict) and between the two types of work-home practices. Our results nuance the dichotomy between users and non-users of work-home practices that has been dominantly used in the work-home practice literature to date and point to similar predictors of work-home conflict among both the group of users and the group of non-users. These findings may encourage researchers to examine characteristics of employees’ work-home practice use (e.g., volition, perceived pressure) in addition to the mere use of these practices when studying their effectiveness.

## Introduction

Due to increased female labor market participation, the rise of single-parent and dual-earner families and changing gender norms (Hammer et al., 2002; Neal and Hammer, 2007; Kossek and Ruderman, 2012; Butts et al., 2013), a growing number of employees today has to combine work with other life roles (Greenhaus and Powell, 2003; Kalliath and Brough, 2008). In response to this new reality, organizations increasingly offer work-home practices to their employees (Thompson et al., 1999; Beauregard and Henry, 2009); i.e., practices which provide additional resources (i.e., flexibility or time) to employees to facilitate balancing their different life roles (Kossek et al., 2010). Work-home practices typically include flexible work arrangements (e.g., home-based telework) and work-time reductions (e.g., part-time work).

Despite the widespread expectation that employees who make use of work-home practices will experience less work-home conflict (Beauregard and Henry, 2009), extant research on the relationship between work-home practice use and both work-to-home conflict (i.e., work interfering with private life) and home-to-work conflict (i.e., private life interfering with work) shows inconsistent results (e.g., Shockley and Allen, 2007; Demerouti et al., 2014; for a meta-analysis, see Kelly et al., 2008). Some studies found, as expected, that employees who make use of work-home practices experience less work-to-home conflict and/or less home-to-work conflict (Byron, 2005; e.g., Hammer et al., 1997; Anderson et al., 2002; Madsen, 2003). However, others studies found no link between these constructs (Mesmer-Magnus and Viswesvaran, 2006; Beauregard and Henry, 2009; e.g., Henz and Mills, 2014) and still others even found these practices to increase work-home conflict (Glass and Finley, 2002; Hill et al., 2003; Hammer et al., 2005; Hilbrecht et al., 2008; Schieman and Young, 2010). Furthermore, if studies find effects of work-home practice use on work-home conflict, effect sizes are generally very small (Gajendran and Harrison, 2007; Allen et al., 2013).

The observed inconsistencies in outcomes of work-home practice use lie at the core of our study. To date, research on the effects of work-home practice use has mainly focused on how users differ from non-users in terms of work-to-home and home-to-work conflict. In doing so, these studies ignore important differences *within* the groups of users and non-users. In this study, we posit that there are similar differences among users and non-users, that are more crucial for understanding work-home conflict than the mere use of work-home practices. We focus on two specific differences: (1) the degree to which employees’ (non-)use of a specific work-home practice is in line with their preference (i.e., volition); and (2) the pressure they experience from the work and/or the home environment to act in another way that they prefer (i.e., perceived work pressure and perceived home pressure). These two characteristics are regularly referred to when researchers try to explain why work-home practice use is sometimes more and sometimes less effective (e.g., Shockley and Allen, 2007; Virick et al., 2010; Delanoeije et al., 2019); however, to the best of our knowledge, no study to date has examined the relevance of these characteristics directly.

We tested the relationship between volition and perceived pressure related to work-home practice use on the one hand and work-to-home conflict and home-to-work conflict on the other hand in two studies: a field study–using survey data with a sample of 381 employees from a middle-large Belgian university–and an experimental vignette study–using a between-subject design with a sample of 556 Belgian employees. In addition, since it has been argued that different practices serve different functions and should therefore be studied separately (Saltzstein et al., 2001; Glass and Finley, 2002; Shockley and Allen, 2007; Kelly et al., 2008), we tested the relevance of volition and perceived pressure for two specific work-home practices, i.e., home-based telework–from now on referred to as telework–and part-time work.

The contribution of our study is fourfold. First, our study extends the dichotomous classification between users and non-users of work-home practices by incorporating important differences within the groups of users and non-users: (1) volition and (2) perceived (work and home) pressure. More insight in this matter may both help to understand current inconsistencies in the literature on work-home practice use and could be useful for organizations to optimize their work-home policies. If employees’ work-home conflict depends on volition and perceived pressure related to work-home practice use, organizations might profit from tailor-made support programs that enable employees to make volitional decisions on work-home practice use, and/or to manage external pressure they experience. Second, by distinguishing between volition and perceived pressure, we acknowledge that both alignment between one’s preference and one’s actual situation (i.e., volition) and fit between one’s preference and environmental expectations (pressure) can affect individuals’ work-home conflict. Third, by testing the relevance of these characteristics not only in a field study, but also in an experimental vignette study, we are better able to link observed effects to our focus variables (i.e., volition, perceived pressure and use). Whereas correlational field studies, which are used most often in research on this topic (Baltes et al., 1999; Butts et al., 2013), are unable to rule out reversed causation or selection effects, vignette studies allow to attribute causality to the factors that are manipulated (in our study: volition, perceived pressure and use), thus precluding reversed causality by design. Fourth, by testing the relevance of volition and perceived pressure for both telework and part-time work, our study includes an immediate replication among two different work-home practices, which can strengthen the conclusions from this study.

## Theoretical Foundations

### Work-Home Conflict

Greenhaus and Beutell (1985) defined work-home conflict as a type of inter-role conflict in which the role demands stemming from one life domain (work or home) are incompatible with role demands stemming from the other domain. The direction of this conflict can go two ways: either individuals can be hindered to meet role demands in the private life due to work demands (“work-to-home conflict”), or they can be hindered to meet role demands in the work domain due to private life demands (“home-to-work conflict”). Previous research has shown work-to-home conflict and home-to-work conflict to be related yet distinct constructs (Kelloway et al., 1999; Byron, 2005).

Work-home conflict generally arises when the time devoted to one role precludes meeting the demands in the other role (time-based conflict) or when stress or strain in one role precludes meeting the demands in the other role (strain-based conflict) (Greenhaus and Beutell, 1985). A third form of conflict (i.e., behavior-based conflict) involves the conflict that arises when the behavior required in one role makes it difficult to fulfill requirements of another role (Greenhaus and Beutell, 1985; for instance, a dominant way of communicating may be effective to reach certain work goals but may not be successful in one’s family life). In this paper, we focus on time-based and strain-based work-home conflict, which we see as the overall affective experience resulting from stress and negative emotions (such as anxiety, irritability and guilt) related to the work-home interface (Greenhaus and Beutell, 1985; Eby et al., 2005; Morgan and King, 2012).

### Work-Home Practice Use and Work-Home Conflict

Since work-home practices offer employees additional resources (e.g., flexibility, time), it is widely expected that using these practices can help employees to lower their work-to-home and home-to-work conflict (Beauregard and Henry, 2009). Yet, as was mentioned above, research on the topic to date has found inconsistent results (Kelly et al., 2008). These inconsistencies stimulated researchers to examine the link between the use of work-home practices and work-home conflict in a more fine-grained way. Research to date has done this in two main ways.

First, to better understand the observed inconsistencies, research started to distinguish between specific work-home practices since different practices serve different functions and may therefore have different effects. These studies indeed found that effects may differ depending on the specific work-home practice (Saltzstein et al., 2001; Glass and Finley, 2002; Shockley and Allen, 2007; Kelly et al., 2008; Allen et al., 2013). Therefore, in the current study, we study the effects of two specific practices that provide different resources to employees, in particular telework (providing additional flexibility) and part-time work (providing additional time).

Second, the inconsistencies in research on work-home practice use also stimulated researchers to examine the role of moderating factors, like home demands (e.g., Golden et al., 2006; ten Brummelhuis and Van der Lippe, 2010), gender (e.g., Greenhaus and Parasuraman, 1999; Sullivan and Lewis, 2001), supervisor support (e.g., Shockley and Allen, 2007; Wang and Walumbwa, 2007), leadership style (e.g., Wang and Walumbwa, 2007), and boundary management preferences (e.g., Delanoeije et al., 2019). Two arguments are frequently used when substantiating the moderation hypotheses. First, several studies focused on moderators that may affect employees’ preference for work-home practice use, contending that work-home practice use will be more effective when this use is more in line with employees’ preference. For instance, Shockley and Allen (2007) argued that work-home practice use would be more effective for employees with high home demands because these employees are more likely to prefer additional resources; and Delanoeije et al. (2019) as well as Virick et al. (2010) expected that working from home would be more effective for employees who preferred integrated work-home boundaries. Second, a number of studies focused on contextual work or home characteristics that are likely to undermine the benefits of work-home practice use and could in that way exert pressure on employees act in another way than they prefer. An example is the study of Shockley and Allen (2007), in which the authors hypothesized less positive outcomes of using work-home practices in organizations that are little family-supportive because users are then likely to experience disapproving sentiments from supervisors and coworkers and a general feeling that use is unacceptable. Similarly, the outcomes of using telework are assumed to be lower when the home environment undermines focus and concentration, for instance when the household size is large (e.g., Golden et al., 2006; Greer and Payne, 2014).

Intriguingly, most of these studies look for moderators that explain variance within the group of users of work-home practices (e.g., Saltzstein et al., 2001; Hammer et al., 2005; Shockley and Allen, 2007). In that way, they ignore that there may also be important–and quite similar–differences among the group of non-users. For instance, in some cases, not using a specific practice may be highly in line with a person’s preference (e.g., when employees do not have caring responsibilities; or when they prefer to maximize their work-related social contacts) and, as such, for these employees, not using a work-home practice may be experienced as highly volitional. Similarly, employees who do not use work-home practices may under certain circumstances experience pressure to make use of a specific practice, for instance when the organization is reducing office space or when the spouse expects the employee to take up more family responsibilities. These differences among non-users have received little research attention to date. This could explain why research results on the role of moderating factors to date are far from conclusive. For example, whereas some studies found that work-home practice use is related with lower work-home conflict when employees experience high home demands/responsibilities (e.g., Byron, 2005; Butts et al., 2013; for a meta-analysis, see Allen et al., 2013), other studies found the opposite effect (e.g., Saltzstein et al., 2001; Hilbrecht et al., 2008; ten Brummelhuis and Van der Lippe, 2010). Also, whereas some studies have shown that women benefit more from work-home practices than men (e.g., Kossek et al., 2006), some studies show the opposite (e.g., Meeussen et al., 2018).

We contend that before looking at moderating factors, it is important to understand the differences among both the group of users and the group of non-users. In line with the arguments frequently used in research on moderating factors, we focus on the following two differences: (1) differences in the degree to which employees’ (non-)use of a specific work-home practice is in line with what they would preferably do (i.e., volition), and (2) in the extent to which employees experience pressure from either the work environment or from their private life to act in a different way than they prefer (i.e., perceived work pressure and perceived home pressure). We posit that both users and non-users vary on these characteristics and that these characteristics are more important for understanding employees’ work-to-home and home-to-work conflict than the mere use of work-home practices. Since people’s use of work-home practices affects how they manage their work and home boundaries, it is likely that work-home conflict may arise when people’ use of these practices is not volitional (so, when the way they manage their work-home boundaries is not in line with their preferences) and/or when they experience pressure to act in another way than they prefer.

Volition and perceived pressure, each in a different way, relate to known concepts from boundary management literature (e.g., Rothbard et al., 2005; Kreiner, 2006; Chen et al., 2009). In particular, volition shows similarities with the concept of boundary fit (i.e., the alignment of individual boundary management preferences with individual boundary management enactments; Ammons, 2008) and perceived pressure aligns with the concept of boundary incongruence (i.e., the misalignment of individual boundary management preferences with environmental boundary management supplies or expectations; Kreiner, 2006). Even in situations of individual fit between desired and enacted behaviors (or, as we label it: volition), employees may still experience boundary incongruence (or, in our terms: pressure) due to different supplies or expectations from their environment. In what follows, we explain volition and perceived pressure related to work-home practice in more detail and argue how these characteristics may affect work-home conflict among both the group of users and non-users.

### Volition

Volition refers to the degree to which employees use or not use a specific work-home practice because they prefer to do so. Individuals are likely to experience volition when they are in a situation that is in line with their preferences because the behavior is then more congruent with their goals and identities (Gagné and Deci, 2005). To date, volition has–to the best of our knowledge–not yet been included in research on the effects of work-home practices. However, there are several indications in the literature that employees differ in their preferences for specific work-home practices and thus in the extent to which they are likely to experience their use or non-use of a specific work-home practice as volitional. For instance, research on work-home boundary management styles has shown that employees differ in their preference to either segment or integrate boundaries between work and private life (Kossek et al., 2012). As telework risks to blur the boundaries between work and home (Ashforth et al., 2000), it seems likely that some employees may prefer to make use of telework while others would preferably not do so. Similarly, research has shown that employees differ in the numbers of hours they preferably work (e.g., Lu, 2011) and, accordingly, it is likely that some employees may prefer to work part-time while others may prefer to work full-time. Accordingly, several scholars have called for the inclusion of individuals’ preferences for telework (Standen et al., 1999) and part-time work (Nardone, 1986; Feldman, 1990) to better understand the effects of these work-home practices.

In this study, we expect that employees who experience their use of a specific work-home practice as volitional, have more positive emotions and less stress and therefore experience less work-home conflict. Volition, or fit between individuals’ behaviors and their preferences, is a central element in several psychological and decision-making theories explaining individuals’ well-being and stress, such as the demand-discretion model (Karasek, 1979), self-determination theory (Deci and Vansteenkiste, 2004; Gagné and Deci, 2005) and decision-justification theory (Connolly and Zeelenberg, 2002). When employees experience a certain choice (e.g., their use or non-use of a specific work-home practice) as volitional, they are likely to feel energized (Lu, 2011), intrinsically motivated (Deci and Vansteenkiste, 2004; Gagné and Deci, 2005) and well able to justify their situation (Connolly and Zeelenberg, 2002), which may all trigger positive emotions and reduce stress (Gagné and Deci, 2005; Kristof-Brown and Jansen, 2007; Boon et al., 2011; Lu, 2011; Kossek and Ruderman, 2012; Verbruggen and van Emmerik, 2018). Reduced stress has, in turn, been related with lower work-home conflict (Burke, 1988; Ilies et al., 2007; for a meta-analysis, see Williams and Alliger, 1994). Conversely, when employees make use of a work-home practice even though they would preferably not do so or, conversely, when they do not make use of a work-home practice while they would preferable do so, they are likely to experience more negative emotions and stress which may then intensify their work-home conflict. In particular, in such cases of low volition, people are managing their work-home boundaries in a way that is not in line with their preference and, consequently, they may experience incongruence between their work and home domain (Edwards and Rothbard, 1999), which may induce stress or strain (Greenhaus and Beutell, 1985). This stress may interfere with one’s abilities to address the demands of the different life domains, thus increasing work-home conflict (Padhi and Pattnaik, 2014). For instance, the stress may increase ruminating over the discrepancy, which consumes energy and would leave less time to fulfill particular domain demands (Greenhaus and Beutell, 1985; Padhi and Pattnaik, 2014).

A few studies support the relevance of volition for work-home conflict. For instance, Gadeyne et al. (2018) showed that work-related ICT-use outside working hours increased work-home conflict for employees with a segmentation preference but not for employees with an integration preference (Gadeyne et al., 2018). Relatedly, Delanoeije et al. (2019) found that employees’ preference to protect their home domain from work intrusions worsened the work-home conflict increasing effect of daily home-to-work boundary role transitions. Similarly, Bogaerts et al. (2018) found a strong negative relationship between perceived boundary management fit and work-home conflict and Lu (2011) showed that a fit between actual and preferred working hours was negatively related with work-home conflict.

Building on the above, we hypothesize:

Hypothesis 1: The degree of volition for using or not using telework is negatively related to work-to-home conflict (Hypothesis 1a) and home-to-work conflict (Hypothesis 1b).

Hypothesis 2: The degree of volition for using or not using part-time work is negatively related to work-to-home conflict (Hypothesis 2a) and home-to-work conflict (Hypothesis 2b).

### Perceived Pressure

It is widely known that constraints from the social context, both at work and at home, can induce pressure upon employees to act in another way than they want to Poelmans (2005). We pose that also in the context of work-home practices, employees may perceive external pressure to act differently than they would preferably do. Although research on work-home practices has rarely included external pressure explicitly, there are several indications in the literature of their existence. Both the work environment and the private life have been repeatedly identified as contexts from which pressure can arise.

First, several studies have pointed to the existence of pressure from the *work environment*, especially pressure to *not* make use of work-home practices. For example, the supervisor or colleagues may induce pressure to not make use of work-home practices, for example when they show little understanding for family issues (Thompson et al., 1999) or, when they view employees’ use of these practices as complicating the work organization (Ilgen et al., 2005; Lembrechts et al., 2018). Accordingly, a family-*un*friendly organizational culture has been suggested to induce perceived pressure to not make use of these practices (Thompson et al., 1999; Anderson et al., 2002; Behson, 2005; Ryan and Kossek, 2008; Kossek et al., 2010). The work environment could also induce pressure to *make* use of offered work-home practices, although this possibility has been mentioned less often in the literature. An indirect reference to this possibility has been made by Hoffman and Cowan (2008), who argued that organizations exert pressure over their employees by offering work-home practices for their employees. Therefore–by merely offering these practices–organizations may create a norm and in that way induce a perceived pressure to make use of these opportunities. Individuals may also perceive a pressure to use work-home practices when organizations reduce the office space because of cost-winning aspects (Stavrinidis, 1991, in Baruch, 2002; Hill et al., 2003).

Second, several studies indicate that employees may perceive pressure from their *private life* to either use or not use work-home practices. For instance, employees with high family-related demands (such as young children) who would preferably not make use of work-home practices may experience a pressure to make use of these practices to take care of these home demands. The home environment may also pressure employees to *not* use work-home practices. For instance, financial factors might pressure employees to not make use of part-time work (Zabalza et al., 1980; Bielby and Bielby, 1989) and having children may induce a pressure to *not* use telework since employees with children tend to expect more interruptions and less productivity while working at home (ten Brummelhuis and Van der Lippe, 2010; Demerouti et al., 2014).

We expect that people who perceive more external pressure related to their work-home practice use, either from the work or the home environment, will experience more work-home conflict. We expect this positive relationship because interrole conflicts occur specifically when individuals experience conflicting role expectations from others in the surroundings (Kahn et al., 1964; Shockley and Allen, 2007). Hence, by its nature, pressure of others in an individual’s environment trigger conflict. Since pressure related to one’s work-home practice use concern how individuals manage their work and home roles, they are likely to affect individuals’ work-home conflict (Piszczek and Berg, 2014). In addition, individuals have a tendency to evaluate their (work and home) environment against internal standards such as their preferences, desires, values or goals (Lazarus and Folkman, 1984; Padhi and Pattnaik, 2014). When individuals experience external pressure to act in another way than they prefer, they are likely to appraise the environment as a threat (Lazarus and Folkman, 1984; Lazarus, 1991), which may elicit negative emotions such as frustration or guilt, inducing stress and therefore (work-home) conflict (Guendouzi, 2006; Morgan and King, 2012; Bochantin and Cowan, 2016). Also the person-environment fit literature suggests that a perceived mismatch between the environment and one’s personal preferences–like in the case of perceived pressure–may induce negative emotions and stress (Harrison, 1978; Edwards and Rothbard, 1999; Padhi and Pattnaik, 2014). Applying this to the specific case of home-based telework, part-time work and work-home conflict, insufficient supplies from the environment to accord with individuals’ preferences concerning the management of boundary domains will create unfulfilled needs and create tension and conflict between these domains, i.e., work and home (Ammons, 2013). Therefore, and in line with suggestions of Poelmans (2005) and Demerouti et al. (2014), we expect that perceived pressure that arises from the work environment (“work pressure”) and from one’s private life (“home pressure”) are related with more work-home conflict:

Hypothesis 3: Perceived work pressure concerning one’s (non-)use of home-based telework is positively related to work-to-home conflict (Hypothesis 3a) and home-to-work conflict (Hypothesis 3b).

Hypothesis 4: Perceived home pressure concerning one’s (non-)use of home-based telework is positively related to work-to-home conflict (Hypothesis 4a) and home-to-work conflict (Hypothesis 4b).

Hypothesis 5: Perceived work pressure concerning one’s (non-)use of part-time work is positively related to work-to-home conflict (Hypothesis 5a) and home-to-work conflict (Hypothesis 5b).

Hypothesis 6: Perceived home pressure concerning one’s (non-)use of part-time work, is positively related to work-to-home conflict (Hypothesis 6a) and home-to-work conflict (Hypothesis 6b).

We tested the relevance of volition and perceived pressure in two studies. First, we conducted a field study using survey data collected with employees (Study 1). Second, to enable the causal inference between our hypothesized independent variables and outcome variables, we also conducted an experimental vignette study (Study 2).

## Materials and Methods of Study 1

### Sample and Procedure

Survey data were collected with employees of a middle-large Belgian university during the summer of 2015. All academic, administrative and technical staff were approached via e-mail to fill in the online survey. A total of 381 staff members (response rate: 30%) filled out the questionnaire. The majority of the sample was female (59.6%). Respondents were between 20 and 64 years old (*M* = 39.56, *SD* = 11.29) and had between 0 and 6 children living at home (*M* = 1.15, *SD* = 1.14). Furthermore, 212 respondents (55.6%) made use of telework and 98 (25.7%) made use of part-time work. Among those who worked part-time, 20 respondents (20.4% of the part-time working respondents) indicated to have another job outside their part-time job at the university. To avoid confounding effects, we left these respondents out of the analyses for part-time work as we do not know whether their total working time adds up to a full-time job or not.

### Measures

#### Volition

We developed an adaptive four-item scale to measure the degree to which the (non-)use of a specific work-home practice is volitional. The items were adapted to the specific work-home practice (i.e., telework and part-time work) and to the respondent’s actual use of that practice. The four items are: (1) “I make use (/do not make use) of [specific work-home practice] because I truly want it like this”; (2) “I would preferably not make use (/make use) of [specific work-home practice]” (reverse scored); (3) “It is entirely my own decision to make use (/to not make use) of [specific work-home practice]”; (4) “If it was entirely up to me, I would not make use (/make use) of [specific work-home practice]” (reverse scored). Items were rated on a five-point Likert scale (1: Totally disagree – 5: Totally agree). Respondents had to fill in the volition scale twice, once for telework and once for part-time work. We tested the validity and reliability of this scale for both telework and part-time work using two other samples, showing support for the quality of this scale (for detailed information about this validation phase: see Appendix A). In this study, the scale turned out to be reliable for both telework (α = 0.93) and part-time work (α = 0.89).

#### Perceived Pressure

We measured perceived pressure from the work environment (“work pressure”) and perceived pressure from the private life (“home pressure”) using single-item measures based on the measures of Shockley and Allen (2015) for “pressure from work” and “pressure from home.” In particular, to assess perceived work pressure, we asked our respondents to evaluate the following item: “I experience pressure from my work or my employer to make use (/to not make use) of [specific work-home practice]”.^1^ Similarly, to assess perceived home pressure, respondents had to evaluate the statement: “I experience pressure from my private life to make use (/to not make use) of [specific work-home practice]”^1^. Respondents had to evaluate the statements on a scale from 0 (Totally disagree) to 10 (Totally agree). Both the measure for perceived work pressure and the one for perceived home pressure had to be filled in twice, once for telework and once for part-time work.

#### Work-to-Home Conflict

Work-to-home conflict was measured using the six items to measure time-based and strain-based work-to-home conflict of Carlson et al. (2000). The six items were found to reliably assess this construct (α = 0.90). Sample items are “The time I must devote to my job keeps me from participating equally in household” and “I am often so emotionally drained when I get home from work that it prevents me from contributing to my family.” The response scale ranged from 1 (Totally disagree) to 5 (Totally agree).

#### Home-to-Work Conflict

Home-to-work conflict was assessed using the six items to measure time-based and strain-based home-to-work conflict of Carlson et al. (2000). The six items had to be rated on a five-point Likert scale ranging from 1 (Totally disagree) to 5 (Totally agree). Sample items are “The time I spend on family responsibilities often interfere with my work responsibilities” and “Due to stress at home, I am often preoccupied with family matters at work.” The scale was found to be reliable (α = 0.84).

#### Controls

We included control variables that have been hypothesized to influence work-home conflict. In particular, we controlled for gender (0 = man; 1 = woman), age (in years), and number of children because women, older employees, and employees with more care dependent children have been shown to experience more work-home conflict (Madsen, 2003; Byron, 2005; Butts et al., 2013). In addition, we included use of telework (1: yes; 0: no) as a control in the regressions on telework and use of part-time work (1: yes; 0: no) in the regressions on part-time work.

### Analyses

Hierarchical regressions were used to test the hypotheses. In a first step, control variables (i.e., age, gender, number of children, use of the specific work-home practice) were entered (Model 1) and in the second step, our key explanatory variables (i.e., volition and the perceived pressure variables) were added (Model 2). The inclusion of use of the specific work-home practice as a control variable is a central point in our study, as we argue that it is not solely the use of practices, but more importantly volition and perceived pressure related to work-home practice use that are relevant for understanding work-home conflict. Multi-collinearity was checked for all predictors by tolerance analysis. All of the predictors’ tolerance were above the cutoff of 0.10 (ranging between 0.41 and 0.99), suggesting that there is no risk for multicollinearity (Tabachnick and Fidell, 2001).

## Results and Discussion of Study 1

Basic descriptive statistics of the sample, reliability coefficients, and correlations between this study’s variables are shown in Table 1. Table 2 shows an overview of all regression outcomes.

**TABLE 1:** Means, standard deviations, reliability coefficients, and correlations of all study variables in Study 1.

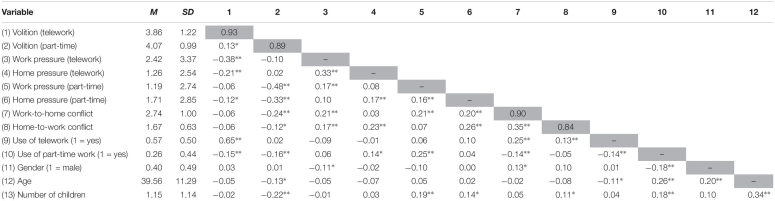

*^∗∗^*p* < 0.01, ^∗^*p* < 0.05. *N* = 381. M = Mean. SD = Standard deviation. Means are on a 1–5 Likert scale, except for work pressure and home pressure (0–10 Likert), use of telework, use of part-time work and gender (dummies), age (years), and children (number). Reliability coefficients are presented on the diagonal axis.*

**TABLE 2:** Standardized regression coefficients (β’s) for effects of controls, volition, perceived work pressure and perceived home pressure in Study 1.

	**Home-based telework^a^**				**Part-time work^b^**			
	**Work-to-home conflict**		**Home-to-work conflict**		**Work-to-home conflict**		**Home-to-work conflict**	
	**Step 1**	**Step 2**	**Step 1**	**Step 2**	**Step 1**	**Step 2**	**Step 1**	**Step 2**
Gender	0.11^∗^	0.14^∗∗^	0.11^∗^	0.12^∗^	0.09	0.10	0.12^∗^	0.12^∗^
Age	–0.05	–0.03	−0.12^∗^	–0.09	–0.04	–0.02	−0.15^∗^	−0.13^∗^
Children	0.06	0.04	0.13^∗^	0.10	0.11	0.03	0.14^∗^	0.10
Use	0.24^∗∗^	0.46^∗∗^	0.12^∗^	0.23^∗∗^	−0.16^∗^	–0.26^∗∗^	0.02	0.01
Volition		–0.32^∗∗^		–0.14		−0.14^∗^		–0.04
Work pressure		0.17^∗∗^		0.09		0.20^∗∗^		0.03
Home pressure		–0.09		0.16^∗∗^		0.13^∗^		0.24^∗∗^
*R*^2^	0.08^∗∗^	0.18^∗∗^	0.05^∗∗^	0.12^∗∗^	0.05^∗^	0.16^∗∗^	0.04^∗^	0.10^∗∗^
Δ*R*^2^	0.08^∗∗^	0.11^∗∗^	0.05^∗^	0.07^∗∗^	0.05^∗^	0.11^∗∗^	0.04^∗^	0.06^∗∗^
*F*	7.39^∗∗^	11.26^∗∗^	4.57^∗∗^	6.96^∗∗^	4.23^∗^	8.93^∗∗^	3.08^∗^	5.21^∗∗^

*^∗∗^*p* < 0.01, ^∗^*p* < 0.05. Age is mean-centered. Gender is a dummy with 0 = female, 1 = male and use is a dummy with 0 = no use and 1 = use; ^*a*^*N* = 360; ^*b*^*N* = 335.*

### Volition

Hypothesis 1 stated that volitional (non-)use of telework would be negatively related with work-to-home conflict (*H1a*) and home-to-work conflict (*H1b*). As shown in Table 2, volition was found to be negatively related with work-to-home conflict (β = −0.32, *p* < 0.01) but not with home-to-work conflict (β = −0.14, *p* = 0.06). These results support *H1a* but not *H1b*.

Hypothesis 2 stated that volitional (non-)use of part-time work would be associated with less work-to-home conflict (*H2a*) and less home-to-work conflict (*H2b*). As shown in Table 2, volition was found to be negatively related with work-to-home conflict (β = −0.14, *p* < 0.05). This supports hypothesis *H2a*. Since the relationship with home-to-work conflict was not significant (β = −0.04, *p* = 0.60), we have to reject hypothesis *H2b*.

### Perceived Pressure

In line with hypothesis *H3a*, we found a positive relationship between work pressure for (not) using telework and work-to-home conflict (β = 0.17, *p* < 0.01). However, no significant relation was found with home-to-work conflict (β = 0.09, *p* = 0.12), so we have to reject *H3b*. Next, unlike hypothesized in hypothesis *H4a*, we did not find a relationship between home pressure for (not) using telework and work-to-home conflict (β = −0.09, *p* = 0.07). Thus, respondents who experienced more home pressure did not report higher work-to-home conflict. We did find the expected positive relationship between home pressure for (not) using telework on home-to-work conflict (β = 0.16, *p* < 0.01), which supports hypothesis *H4b*.

In line with hypothesis *H5a*, we found a positive relationship between work pressure for (not) using part-time work and work-to-home conflict (β = 0.20, *p* < 0.01). Yet, we did not find a relationship with home-to-work conflict (β = 0.03, *p* = 0.65) and can thus not support hypothesis *H5b.* In line with hypotheses *H6a* and *H6b*, home pressure for (not) using of part-time work was found to be positively related with both work-to-home conflict (β = 0.13, *p* < 0.05) and home-to-work conflict (β = 0.24, *p* < 0.01).

### Volition and Perceived Pressure Versus Use

Finally, we had a look at the impact of the use of telework and the use of part-time work. In our theorizing, we posited that volition and perceived pressure related to work-home practice use would be more crucial for understanding work-home conflict than the mere use of specific work-home practices. To evaluate this assumption, we compared the explained variance of Model 1 (in which the mere effect of practice use was examined, as done in traditional studies investigating the effect of practice use) with the explained variance of Model 2. For telework, the inclusion of volition and perceived pressure was found to more than double the explained variance of work-to-home conflict and home-to-work conflict compared to Model 1. Similarly, for part-time work, the inclusion of volition and perceived pressure was found to increase the explained variance of work-to-home conflict and home-to-work conflict up to three times. So, in line with our expectations, volition and perceived pressure seemed to be more important than the mere use of work-home practices in explaining work-home conflict.

### Discussion

The results of this survey study suggest that volition, perceived work pressure and perceived home pressure are all relevant for understanding employees’ work-home conflict, yet, these factors seem to be more important for understanding work-to-home conflict than for home-to-work conflict.

First, in line with our expectations, *work-to-home conflict* was found to be related with volition and perceived work pressure in both the regression on telework and the regression on part-time work, as well as with perceived home pressure in the regression on part-time work. However, unlike hypothesized, perceived home pressure related to telework was not linked with higher work-to-home conflict. It could be that there is a compensatory reversed causation effect and that employees with low work-to-home conflict experience more home pressure, for instance from their spouse, to use telework since the work-home combination is now going “so easy” for them and using telework could then enable them to take up more home responsibilities additive to their work role. We cannot examine this reversed causation path due to the cross-sectional nature of the data.

Second, for *home-to-work conflict*, we only found a significant link with perceived home pressure (in both the regression on telework and the regression on part-time work). Neither volition, nor perceived work pressure were found to be related with home-to-work conflict. Perhaps, floor effects may inhibited us to observe an impact, since participants scored at the lower end of the home-to-work conflict. Alternatively, it could be that volition and perceived work pressure are not so relevant for understanding variation in home-to-work conflict. Perhaps, involuntary interruptions from the private life may still cause home-to-work conflict (Matthews et al., 2010; Carlson et al., 2015; Smit et al., 2016), irrespective of whether employees’ use of a specific work-home practice is volitional or of whether employees perceive pressure from the work context to act in another way than they prefer. It could also be that there are compensatory effects at play. For instance, employees who perceive high work pressure can perhaps adapt their private life role (for instance, adapt the way their private life is organized, such as getting household care to lower home demands) to that extent that their private life does not further interfere with their work, which may buffer the expected positive effect of work pressure on home-to-work conflict.

Interestingly, our results suggest that home pressure is related with both work-to-home and home-to-work conflict, whereas work-pressure is only relevant for understanding work-to-home conflict. This is in line with earlier research on private life and work stressors, which has shown that private life stressors affected both work-to-home and home-to-work-conflict, whereas job stressors affected work-to-home conflict to a greater extent than it affected home-to-work conflict (Byron, 2005).

Overall, our findings show that volition and perceived pressure explained more variance than the mere use of a specific work-home practice. These results support our argument to include these factors when studying the effect of work-home practices on work-home conflict. A major limitation of this study is that we used cross-sectional data. In addition, for some of our explanatory variables, mainly for volitional (non-)use of part-time work, we found a high mean and low variance, which may have lowered the likelihood of observing an effect of this variable due to ceiling effects. To strengthen our findings, to test the hypothesized causal direction between the variables and avoid problems related to ceiling effects in our explanatory variables, we also tested the relevance of all characteristics (i.e., volition, perceived work pressure and perceived home pressure) in an experimental vignette study.

## Materials and Methods of Study 2

### Sample and Procedure

Data were collected using snowball sampling in the spring of 2016. Belgian employees were recruited through a call for participation sent out by one postgraduate student and eight undergraduate students from different regions in the country to increase geographical distribution. We targeted employees who had been working for at least 6 months to assure that respondents were familiar with working in an organization and would be able to understand and reliably assess the vignettes (Wason et al., 2002; Aguinis and Bradley, 2014). In total, 556 employees filled in this experimental survey. Sixty-three percent of the respondents were female respondents. Respondents were between 21 and 51 years old (*M* = 34.98, *SD* = 9.53) and had between 0 and 5 children (*M* = 1.12, *SD* = 1.24). Furthermore, 37.1% of the respondents made use of telework and 23.7% made use of part-time work.

We presented each respondent with two short stories, one related to telework and one related to part-time work. Half of the respondents was first presented the scenario on telework and the other half received the scenario on part-time work first. In both stories, we manipulated volition, perceived work pressure, perceived home pressure and actual use of the work-home practice, resulting in a randomized 2 (volition: yes/no) by 2 (perceived work pressure: yes/no) by 2 (perceived home pressure: yes/no) by 2 (use: yes/no) design. This resulted in a total of 16 experimental conditions, which were presented *between* (rather than within) respondents to avert potential fatigue (Weber, 1992). Cell sizes for this 2 × 2 × 2 × 2 design ranged between 31 and 42 (*N* = 556).

### Vignettes

In line with recommendations of Aguinis and Bradley (2014), we presented all respondents the same baseline information to allow for comparison between respondents. Respondents were first informed that we would present them two hypothetical stories related to work-home practices, described as practices that organizations can offer to facilitate employees’ combination of work with private life. Then two vignettes were presented.

The telework vignette described an employee who has two school-going children and works for an organization that offers the option to make use of telework, defined as the possibility to work from home on work-related tasks during regular work hours. Similarly, the part-time work vignette described an employee with two school-going children who works for an organization that offers the option to work part-time, defined as the option to work less hours than a full-time job, for example 60%. We specified that the employee had two school-going children in order to make the two stories more comprehensible (Aguinis and Bradley, 2014) and to control for the influence of care-dependent children, which is known to affect work-home conflict (Byron, 2005; Shockley and Allen, 2007). The rest of the scenario was adapted to the different experimental manipulations. A sample vignette for the condition of volition (yes), perceived work pressure (yes), perceived home pressure (yes), and use of part-time work (yes) is the following:

Imagine an employee in an organization. This organization offers the option to work part-time, i.e., the option to work less hours than a full-time job, for example 60%. This employee has two school-going children and has a personal preference to work part-time. Thus, if it was completely up to this person, he/she would work part-time. This employee also works part-time. However, this employee experiences pressure to work full-time from his/her supervisor as well as from his/her partner.

After each scenario, respondents were asked to assess the work-to-home conflict and home-to-work conflict of the employee in the story. Given that participants rated another individual’s work-to-home conflict, we indicated participants to keep in mind the situation of the employee described in the scenario.

Rather than asking respondents to imagine themselves as an employee with certain fixed (e.g., having two school-going children) or manipulated characteristics (e.g., having a preference to work part-time, experiencing pressure from supervisor and/or partner to work part-time), we asked employees to imagine *another* employee and rate the expected amount of work-home conflict they would think this other employee would experience. In this way, we aimed to limit bias from respondents’ own background characteristics (e.g., number of children) or respondents’ own levels of volition, perceived pressure and work-home conflict and, hence, to study the pure effects of the *manipulated* independent variables since we were not interested in the effects of respondents’ own background information.

### Measures

#### Volition

High volition (coded 1) was manipulated by stating that the employee’s use or non-use of the specific work-home practice is in line with what this employee would choose to do if it was entirely up to this employee him- or herself. Similarly, low volition (coded 0) was manipulated by stating that the employee’s use or non-use of the specific work-home practice was the opposite of what the employee would choose if it was entirely up to this employee him- or herself.

#### Perceived Work Pressure

Perceived work pressure was manipulated by stipulating that the employee perceived (coded 1) or did not perceive (coded 0) pressure from his/her supervisor to do the opposite of what (s)he preferred to do if it was entirely up to him- or herself. In line with Greenhaus and Powell’s (2003) vignette manipulation for perceived work pressure, we narrowed down the work environment to one aspect, i.e., the supervisor, to make interpretation of the vignette easier for respondents (Aguinis and Bradley, 2014). We specifically choose for the supervisor as supervisor support has been consistently found to affect work-home conflict (e.g., Frye and Breaugh, 2004). A manipulation check, which asked the respondents to what extent the described employee experienced pressure from his/her supervisor (1: ‘No pressure at all’ – 7: ‘A lot of pressure’), showed that, as intended, the respondents reported significantly more pressure in the ‘work pressure’ condition than in the ‘no work pressure’ condition (*F*(1,547) = 402.12, *p* < 0.01, for vignettes on telework; *F*(1,546) = 505.12, *p* < 0.01, for vignettes on part-time work).

#### Perceived Home Pressure

Similarly, home pressure was manipulated by stipulating that the employee perceived (coded 1) or did not perceive (coded 0) pressure from his/her partner to do the opposite of what (s)he would prefer to do if it was entirely up to him- or herself. In line with Greenhaus and Powell’s (2003) vignette manipulation for perceived home pressure, we narrowed down the private environment to one aspect, i.e., the partner, to make interpretation of the vignette easier for respondents (Aguinis and Bradley, 2014). We specifically choose for the partner as work-home decisions are often made at a couple level (Moen and Yu, 2000) and work-home practice use has shown to have cross-over effects on work-home conflict (Schooreel and Verbruggen, 2015). A manipulation check, which asked respondents to what extent the employee in the vignette experienced pressure from his/her partner (1: ‘No pressure at all’ – 7: ‘A lot of pressure’), showed that in line with our manipulation, respondents reported significantly more pressure in the ‘home pressure’ condition than in the ‘no home pressure’ condition (*F*(1,547) = 402.12, *p* < 0.01, for vignettes on telework; *F*(1,546) = 505.12, *p* < 0.01, for vignettes on part-time work).

#### Use

The use or non-use of part-time work and telework was manipulated by specifying whether the employee made use (coded 1) or did not make use (coded 0) of the specific work-home practice.

#### Work-to-Home Conflict

For work-to-home conflict, the statements we used were slight modifications of the six items of Carlson et al. (2000) as we used in the survey data. Sample items are “The work of this employee keeps him/her from his/her family activities more than (s)he would like” and “When this employee gets home from work (s)he is often too frazzled to participate in family activities/responsibilities.” The response scale ranged from 1 (Totally disagree) to 5 (Totally agree). This scale was found to be reliable in both the scenarios on telework (α = 0.90) and on part-time work (α = 0.93).

#### Home-to-Work Conflict

For home-to-work conflict, we opted to use an adapted single-item measure to decrease respondent fatigue. In particular, we directly asked how respondents assessed the home-to-work conflict by the following question: “All in all, to what extent do you think that this employee is experiencing a negative influence from his/her private life on his/her work?” Respondents could answer this question on a scale from 1 (No negative influence at all) to 7 (Very strong negative influence). We consider this adapted single-item scale reliable as we found a similar single-item measure for work-to-home conflict (i.e., “All in all, to what extent do you think that this employee is experiencing a negative influence from his/her work on his/her private life?”) to correlate highly with the validated full six-item scale for work-to-home conflict for both the scenario’s on telework (*r* = 0.61, *p* < 0.01) and on part-time work (*r* = 0.64, *p* < 0.01).

### Analyses

We conducted two-way analyses of variance to examine the influence of three key predictors (volition, work pressure, home pressure) on work-to-home and home-to-work conflict. In all analyses, we controlled for the use of the specific work-home practice.

## Results and Discussion of Study 2

Table 3 shows an overview of all outcomes of the analyses of variance.

**TABLE 3:** Means of work-to-home conflict and home-to-work conflict and analyses of variance for variables of use, volition, work pressure and home pressure in Study 2.

	**Home-based telework**						**Part-time work**					
	**Work-to-home conflict**			**Home-to-work conflict**			**Work-to-home conflict**			**Home-to-work conflict**		
	**(*M* = 2.55, *SD* = 0.81)**			**(*M* = 3.32, *SD* = 1.43)**			**(*M* = 2.64, *SD* = 1.00)**			**(*M* = 3.40, *SD* = 1.47)**		
	***F***	***MS***	***MD***	***F***	***MS***	***MD***	***F***	***MS***	***MD***	***F***	***MS***	***MD***
Use	0.64	0.40	−0.05(0.07)	1.24	2.32	0.13(0.12)	73.19^∗∗^	62.94	−0.67^∗∗^(0.08)	4.58^∗^	9.09	−0.25^∗^(0.12)
Volition	15.04^∗∗^	9.23	−0.25^∗∗^(0.07)	1.66	3.10	−0.15(0.12)	5.48^∗^	4.72	−0.19^∗^(0.08)	17.52^∗∗^	34.80	−0.51^∗∗^(0.12)
Work pressure	23.71^∗∗^	14.55	0.33^∗∗^(0.07)	0.44	0.82	0.08(0.12)	12.24^∗∗^	10.53	0.28^∗∗^(0.08)	0.12	0.23	0.05(0.12)
Home pressure	8.71^∗∗^	5.34	0.20^∗∗^(0.07)	53.81^∗∗^	100.30	0.86^∗∗^(0.12)	12.38^∗∗^	10.62	0.28^∗∗^(0.08)	33.23^∗∗^	66.00	0.70^∗∗^(0.12)
Error	550	0.61		539	1.86		551	0.86		541	1.99	
*R*^2^	0.08		0.10			0.16			0.09			

**N* = 556. ^∗∗^*p* < 0.01 ^∗^*p* < 0.05. *M* = mean. *SD* = standard deviation. *F* = *F*-statistic. MS = the mean sum of squares due to the focus variable. *MD* = mean difference using pairwise comparisons between 0 and 1 categories of variables, based on estimated marginal means. Standard errors are given in parentheses. The means of work-to-home conflict are on a 1–5 Likert scale, the means of home-to-work conflict are on a 1–7 Likert scale.*

### Volition

In line with hypothesis *H1a*, volitional (non-)use of telework was negatively related with work-to-home conflict (*F*(1,548) = 14.50, *p* < 0.01). However, contrary to expectation (*H1b*), volitional (non-)use of telework was not significantly related with home-to-work conflict (*F*(1,537) = 1.54, *p* = 0.22). In line with hypotheses *H2a* and *H2b*, volitional (non-)use of part-time work was negatively related with both work-to-home conflict (*F*(1,549) = 5.62, *p* < 0.05) and home-to-work conflict (*F*(1,539) = 17.79, *p* < 0.01).

### Perceived Pressure

Hypotheses H3 and H4 related to telework. In line with hypothesis *H3a*, work pressure for (not) using telework was positively associated with work-to-home conflict (*F*(1,548) = 23.84, *p* < 0.01). The expected association with home-to-work conflict was, however, not found (*F*(1,537) = 1.54, *p* = 0.22) and we thus have to reject hypothesis *H3b*. In line with hypotheses H4a and H4b, we found the expected positive relationship between home pressure for (not) using telework and both work-to-home conflict (*F*(1,548) = 8.67, *p* < 0.01) and home-to-work conflict (*F*(1,537) = 54.13, *p* < 0.01).

Hypotheses H5 and H6 related to part-time work. In line with hypothesis *H5a*, we found the expected positive relationship between work pressure for (not) using part-time work and work-to-home conflict (*F*(1,549) = 12.35, *p* < 0.01), indicating less work-to-home conflict in the conditions without work pressure (*M* = 2.50, *SD* = 0.06) than in those with work pressure (*M* = 2.77, *SD* = 0.06). The expected positive relationship with home-to-work conflict was, however, not found (*F*(1,539) = 0.15, *p* = 0.70) and we thus have to reject hypothesis *H5b*. For home pressure, we found the expected positive relationship with both work-to-home conflict (*F*(1,549) = 12.48, *p* < 0.01) and home-to-work conflict (*F*(1,539) = 34.28, *p* < 0.01), indicating more work-to-home conflict and more home-to-work conflict in conditions with home pressure than in those without home pressure. This is in line with *H6a* and *H6b*.

### Volition and Perceived Pressure Versus Use

As we expected, volition and perceived pressure explained considerably more variance of work-to-home and home-to-work conflict that the use of the specific work-home practice. For telework, we did not see a significant impact of use on work-to-home conflict or home-to-work conflict whereas volition and the perceived pressure variables together explained 8% of the variance in work-to-home conflict and 10% of the variance in home-to-work conflict (Table 3). For part-time work, volition and perceived pressure were found to increase the explained variance of work-to-home conflict (*R*^2^ = 0.16) and home-to-work conflict (*R*^2^ = 0.09) up to nine times compared to a model including only the use of the specific practice (*R*^2^_work–to–home conflict_ = 0.10; *R*^2^_home–to–work conflict_ = 0.01) (Table 3). This supports our expectation that these characteristics are more important than mere use for understanding differences in work-home conflict.

### Discussion

As expected, we found all characteristics (i.e., volition, perceived work pressure and perceived home pressure) to be linked with *work-to-home conflict* in the expected direction for both telework and part-time work. Thus, contrary to Study 1, we did find an effect of home pressure for (not) using telework. This may support our reversed causation explanation we gave earlier for the null-effect in Study 1; i.e., that our hypothesized positive relationship between home pressure and work-to-home conflict may be countered by a compensatory reversed causation effect implying a negative relationship between work-to-home conflict and home pressure (i.e., perhaps employees with low work-to-home conflict experience more pressure from their spouse to take up more home responsibilities since things are going so easy for them). Indeed, the results of this experimental vignette study are by design less prone to a reversed direction of causality.

As in Study 1, *home-to-work conflict* was not affected to the same extent as work-to-home conflict by volition and perceived pressure. For telework, we found the expected negative effect of home pressure on home-to-work conflict, yet – like in Study 1 – neither volition nor work pressure affected home-to-work conflict. For part-time work, both volition and home pressure were linked with home-to-work conflict in the expected direction, but again, work pressure did not affect home-to-work conflict. These findings for home-to-work conflict were thus largely the same as in Study 1, except for one difference: whereas volitional (non-)use of part-time work was not related to home-to-work conflict in study 1, we did find a significant relationship in Study 2. This may support the explanation we gave earlier for not finding this effect in Study 1, i.e., that this could be due either to the high scores and the low variance of volitional (non-)use of part-time work (i.e., ceiling effects) or to the low scores on the home-to-work conflict scale (i.e., floor effects) in Study 1.

## General Discussion

The results of both Study 1 (field survey) and Study 2 (experimental vignette survey) largely support the main proposition of this paper, i.e., that characteristics of employees’ (non-)use of a specific work-home practice are more important than the mere use of that practice to understand variance in work-home conflict. In particular, the two characteristics we included in this study, i.e., (1) volition for the (non-)use of telework and part-time work, and (2) perceived external pressure from the work context and/or from the private life, were found to explain at least double–and up to three times–the variance in work-home conflict in Study 1 and up to nine times the variance in work-home conflict in Study 2 compared to the mere use of a specific work-home practice.

### Work-to-Home Conflict

Work-to-home conflict was significantly related with all but one of the characteristics of work-home practice use we included–i.e., volition, perceived work pressure and perceived home pressure–in both the regression on telework and the regression on part-time work. We failed to find one effect in Study 1, namely an influence of home pressure in the regression on telework, but as we explained earlier, this is likely due to a compensatory reversed causation effect related to the cross-sectional nature of our dataset in this study.

### Home-to-Work Conflict

Home-to-work conflict, on the other hand, was mainly related with perceived home pressure. In Study 2, we also found a positive relationship with volition in the regression on part-time work. While we failed to find this effect in Study 1, we believe this may be due to the relatively high mean value and low variance of volitional (non-)use of part-time work in that setting, or to the relatively low scores on the home-to-work conflict scale in this study.

In none of our studies, we found the expected effect of volitional (non-)use of telework on home-to-work conflict. Although this lack of effect on home-to-work conflict may also be due to the low scores on this variable, an alternative explanation for this finding may lie in the nature of telework as both when working from home and when working at the office, (in)voluntary interruptions from the private life may occur and cause home-to-work conflict (Allen et al., 2003; Smit et al., 2016) –irrespective of the extent to which one’s (non-)use of telework is volitional. These findings may also indicate that volition has a differential effect on home-to-work conflict depending on whether the volition relates to telework or to part-time work. This supports earlier recommendations to distinguish between specific work-home practices as each practice may function differently and should therefore be studied separately (Saltzstein et al., 2001; Glass and Finley, 2002; Shockley and Allen, 2007; Kelly et al., 2008).

In addition, in both studies, we found that perceived work pressure was not related with home-to-work conflict. Hence, work pressure and home pressure do not seem to affect home-to-work conflict to the same extent, which supports the relevance of distinguishing between different sources of pressure. The importance of this distinction has also been shown in other research domains, like research on embeddedness (Lee et al., 2004) and turnover (Hom et al., 2012). These findings are also in line with earlier suggestions that work and private life may affect work-home conflict differently, and that the private life context should best be included when understanding employees’ work-home conflict (Edwards and Rothbard, 1999; Poelmans, 2005; Padhi and Pattnaik, 2014). Overall, it seems important for future research to examine further why these differences between different sources of external pressure occur and to include these explanations in further theorizing on this issue.

### Theoretical Contributions

Our results first indicate the need for scholars to rethink how we evaluate the effectiveness of work-home practices. To date, studies on work-home practices have indicated that work-home practices are not always used when available (McDonald et al., 2005; Allen et al., 2013), and that, in the case of use, the use is not always associated with the intended positive effects on employee outcomes (e.g., Butts et al., 2013). We argue that not using available practices does not have to indicate a failed implementation policy neither does use of these practices imply a successful implementation. Rather, our results point to the necessity of using an employee-centered approach that focusses on how employees perceive the characteristics of their (non-)use of a specific work-home practice (i.e., volition and perceived external pressure) to evaluate the success of a work-home policy implementation. This suggestion follows up on earlier recommendations to consider the subjective experience of employees rather than to look at objective ciphers of use of work-home practices when studying their effectiveness (Guest and Bos-Nehles, 2013).

Second, although scholars have regularly referred to volition and external pressure when explaining why certain factors may moderate the effectiveness of work-home practice use (Thompson et al., 1999; Sullivan and Lewis, 2001; Golden et al., 2006; Shockley and Allen, 2007; Wang and Walumbwa, 2007), our study examined these characteristics directly, in that way providing a good basis for further moderation studies. By distinguishing between volition and perceived pressure, our study emphasizes the potential difference between employees’ wants and demands and indicated the need for researchers to consider whether certain demands (e.g., home demands, work demands) may feel as volitional (i.e., wants) or either may function as a pressure not in line with these wants. In addition, our study may stimulate researchers not to only pay attention to differences among users of work-home practices, but also among non-users. Most moderation studies on the topic (e.g., Thompson et al., 1999; Golden et al., 2006) have focused primarily on understanding variation in outcomes among users, thereby treating non-users as a homogenous reference group. The lack of attention to variation among non-users is in line with a general tendency in psychological research to focus on factors that motivate or energize organisms to move, change or take action, thereby overlooking variation among those *not* changing or *not* taking action (Anderson, 2003; Verbruggen and De Vos, 2019). Our study showed that–irrespective of employees’ actual use–volition and perceived pressure related to work-home practice (non-)use are important for understanding work-home conflict, which suggests that similar characteristics explain outcomes among both users and non-users.

Third, some differences between the results of Study 1 and Study 2 illustrated the relevance of using experimental designs. Experimental designs rule out alternative explanations such as reversed causality and selection effects by design, which makes them an interesting addition to traditional field studies.

Fourth, our results affirm the need for scholars to study work-home practices separately since we found some differences between effects on telework and on part-time work when studying work-home conflict. However, even among these two different practices, our findings indicate the importance of volition and perceived external pressure for both work-home practices, especially when understanding variation in work-to-home conflict. Overall, our findings are in line with previous recommendations that rather than looking at *objective* measures of the work-home interface (e.g., looking at the specific amount of time or resources to allocate to either the work or the non-work domain, or looking at objective working conditions such as use versus non-use of work-home practices), researchers should consider employee’s *subjective* perceptions as antecedents of work-home conflict (Valcour, 2007; Grawitch et al., 2010, 2011). We consider volition and perceived pressure as (subjective) perceptions related to the (objective) use of work-home practices and our results show that these characteristics indeed matter above and beyond the mere use of work-home practices. In the same perspective, authors have already argued to evaluate work-home practices based on their alignment with characteristics of employees, their perceived work context, and their broader private life context (Grawitch et al., 2011). Our research follows up this recommendation by including volition and perceived pressure from two different life spheres to (not) use work-home practices.

### Limitations and Future Research

Our study has a number of limitations. First, some methodological considerations should be made. In line with earlier studies (e.g., Shockley and Allen, 2015), we used single-item measures to measure perceived pressure. Future research may want to develop and validate multiple item scales to improve the assessments of perceived pressure. Second, we used a single item measure for home-to-work conflict in our vignette study. Whereas we did find the same item adapted for work-to-home conflict to be reliable, we could not validate the single item for home-to-work conflict and therefore, this measure needs to be treated with some caution. Third, in our vignette study, we asked participants to rate another employee’s work-home conflict. Therefore, in this study, we may have gaged to a slightly different variable of work-home conflict, i.e., projected work-home conflict rather than self-experienced conflict. Future research may benefit from replicating our results with an experimental manipulation of self-experienced work-home conflict. Fourth, we used self-report cross-sectional data for our field study and therefore cannot rule out correlational instead of causal effects in this study. Whereas we extended our study results with these of an additional experimental study, future research may is needed to further test directionality in a field setting. This could for instance be done using a longitudinal field study including measurements of volition and perceived pressure at different time points, both *before* and *after* decisions about use and non-use of practices are made and implemented. An additional asset of this approach is that decisions on work-home practice use might then be studied in more detail, which may reveal possible effects of cognitive dissonance and internalization (i.e., becoming satisfied with circumstances as they are and internalize these circumstances as volitional). Fifth, an interesting future pathway may consist out of testing also the effects of perceived pressure *in line with* employees’ preferences. Using a person-environment fit perspective, organizational pressure congruent with an employees’ preference may have different effects than incongruent pressure (Kreiner, 2006). Finally, an interesting pathway for future research may lie in studying specific interactions between use of work-home practices on the one hand and volition and pressure related to use on the other hand. Since we did not find all of our hypothesized relationships to be significant, some effects may be stronger or weaker depending on the specific sample under study (i.e., users versus non-users). Whereas our study provided a first step in establishing the basic relationships between volition and pressure and work-home conflict, future research may add nuances to the main propositions of this paper and identify moderators of the hypothesized relationships.

### Practical Implications

Our results show that for work-home practices to have beneficial effects, employees should be allowed to make use of work-home practices if they want to, without experiencing pressure to either use or not use offered practices. Enabling employees to have control over their use of these practices and not pressuring them thus seems to be key in a successful implementation. Yet, our results also showed that pressure from employees’ private life is predictive for their work-home conflict. Therefore, effective organizational implementation of work-home practices may be insufficient to guarantee low work-home conflict. Career counseling could be one path through which organizations may help their employees to cope with pressure from their private life. For instance, research has shown that employees can benefit from certain psychological techniques to cope with diverging responsibilities from different life roles (Versey, 2015). Finally, if outcomes depend on employees’ volition and perceived pressure, organizations might profit from tailor-made support programs that help employees to reach a match between working conditions and their preferences, enhancing volitional use, and/or to manage perceived external pressure. Enabling employees to make volitional choices and asserting them more control over working conditions might then optimize the effects of work-home practices. Additionally, organizations may consider idiosyncratic employment arrangements (i.e., “i-deals”; Rousseau, 2005) when work-home practices do not fit with an employees’ home context. Research has found alleviating effects of flexibility i-deals on employees’ work-home conflict (Bayazit and Bayazit, 2017) and has in general found positive effects of flexibility i-deals on employee performance (Marescaux et al., 2012) and commitment (Las Heras et al., 2017). Thus, idiosyncratic deals could be one means to align employees’ contextualized wants and obligations and those of the organization.

## Conclusion

In this study, we showed the relevance of including aspects associated with the use or non-use of work-home practices for understanding the effectiveness of these practices. We found evidence that (1) *volition* for use or non-use of telework and part-time work and (2) *perceived pressure* from the work environment and from the private environment to use or not use these practices explained more variance in both work-to-home conflict and home-to-work conflict than the mere use of these work-home practices. We therefore encourage scholars and practitioners to focus on these characteristics rather than on measures of mere use when studying the effectiveness of work-home practices.

## Data Availability Statement

The raw data supporting the conclusions of this manuscript will be made available by the authors, without undue reservation, to any qualified researcher.

## Ethics Statement

Ethical review and approval was not required for the study on human participants in accordance with the local legislation and institutional requirements. The participants provided their written informed consent to participate in this study.

## Author Contributions

JD and MV developed the research idea and conceptual framework of the article and collected the data. JD performed the data analysis, interpreted the results, and drafted the manuscript. MV provided critical revisions and edited the manuscript. Both authors made substantial contributions to the work reported in the article and approved the final version of the manuscript.

## Conflict of Interest

The authors declare that the research was conducted in the absence of any commercial or financial relationships that could be construed as a potential conflict of interest.

## Footnotes

1 Since we hypothesized an impact of the pressure employees experience to act in another way than they prefer, the measurement was adapted based on their score on the volition-measure. In particular, we assessed “pressure to use a specific work-home practice” if employees indicated they would *preferably not use* that practice (i.e., for users: when they had a score of 3 or higher on the volition scale and for non-users: when they had a score lower than 3 on the volition scale) and we assessed “pressure to not use a specific work-home practice” if employees indicated they would *preferably use* that practice (i.e., for users: when they had a score lower than 3 on the volition scale and for non-users: when they had a score of 3 or higher on the volition scale).

## Appendix A: Validation of Volition Scale

We validated the volition scales for telework and part-time work using two additional datasets following guidelines of Netemeyer et al. (2003). An initial validation of the scales was conducted using a sample of 467 working parents recruited through kindergartens and primary schools. Exploratory factor analysis showed that the eight items loaded on two factors and all factor loadings were above 0.78. The corrected item-to-total correlations were all above 0.64, indicating that the items correlated well with the overall scale (Everitt, 2002; Field, 2005). In addition, both the volition scale for telework (α _*home–based telework*_ = 0.90) and the volition scale for part-time work (α _*part–time work*_ = 0.90) showed good internal consistency.

Validity, reliability and construct stability of the scales were further tested in a second sample, i.e., a two-wave online survey study conducted with 118 employees. Respondents filled in two questionnaires, 1 month apart. We first performed a confirmatory factor analysis (CFA) on the four items measuring volition for telework and the four items measuring volition for part-time work using a Satorra–Bentler correction to correct for non-normality (Satorra and Bentler, 2001). Result of this CFA showed support for the expected two-factor structure: one factor capturing volition for telework and one factor capturing volition for part-time work (χ*^2^*[19] = 56.70, *p* < 0.001; SRMR = 0.07; CFI = 0.94; TLI = 0.91; Hoyle, 1995; Hu and Bentler, 1999). All items loaded well on their respective factor (factor loadings ranging between 0.56 and 0.97, *p* < 0.001). Second, reliability analyses showed that both the volition scale for telework and the volition scale for part-time work had good internal consistency at both wave 1 (α _*home–based telework*_ = 0.90; α _*part–time work*_ = 0.89) and at wave 2 (α _*home–based telework*_ = 0.89; α _*part–time work*_ = 0.89). Test-retest reliability was high for the scale on part-time work (*r* = 0.78, *p* < 0.001) and moderate for the scale on home-based telework (*r* = 0.58, *p* < 0.001) (DeVon et al., 2007; Weafer et al., 2013). Finally, we analyzed construct stability over time by testing a model wherein we let indicators at the first test occasion (T1) correlate with their corresponding indicator at the retest occasion (T2). We first checked whether the constructs are measured by the same measurement model at T1 and T2 by comparing a model not assuming measurement invariance (i.e., factor loadings on indicators at T1 and at T2 are allowed to fluctuate freely) with a model assuming measurement invariance (i.e., factor loadings on indicators at T1 and at T2 are set equal). Fixing the factor loadings to be equal at T1 and T2 did not worsen the fit of the model (χ*^2^*diff = 10.77, *p* = 0.10), and this model showed acceptable fit (χ*^2^*[102] = 216.52, *p* < 0.001; SRMR = 0.08; CFI = 0.92; TLI = 0.90), which confirms measurement invariance. We then evaluated construct invariance (i.e., whether the construct mean is stable) by estimating the construct intercepts at T1 and at T2 and see whether they differed from each other. The intercepts did not differ from each other for both the scale for telework (*t*(63) = 1.29, *p* = 0.92) and the scale for part-time work (*t*(63) = 1.56, *p* = 0.94), which confirms stability over time for both constructs.
